# Effectiveness of a structured educational intervention using psychological delivery methods in children and adolescents with poorly controlled type 1 diabetes: a cluster-randomized controlled trial of the CASCADE intervention

**DOI:** 10.1136/bmjdrc-2015-000165

**Published:** 2016-06-01

**Authors:** Deborah Christie, Rebecca Thompson, Mary Sawtell, Elizabeth Allen, John Cairns, Felicity Smith, Elizabeth Jamieson, Katrina Hargreaves, Anne Ingold, Lucy Brooks, Meg Wiggins, Sandy Oliver, Rebecca Jones, Diana Elbourne, Andreia Santos, Ian C K Wong, Simon O'Neil, Vicki Strange, Peter Hindmarsh, Francesca Annan, Russell M Viner

**Affiliations:** 1 University College London Hospitals NHS Foundation Trust, London, UK; 2 Social Sciences Research Unit, Institute of Education, London, UK; 3 London School of Hygiene and Tropical Medicine, London, UK; 4 UCL School of Pharmacy, London, UK; 5 Diabetes UK, London, UK; 6 Royal Liverpool Children's Hospital NHS Trust, Liverpool, UK; 7 UCL Institute of Child Health, London, UK

**Keywords:** Adolescent Diabetes, Education and Behavioral Interventions, Psychology, Randomized Controlled Trial

## Abstract

**Introduction:**

Type 1 diabetes (T1D) in children and adolescents is increasing worldwide with a particular increase in children <5 years. Fewer than 1 in 6 children and adolescents achieve recommended glycated hemoglobin (HbA1c) values.

**Methods:**

A pragmatic, cluster-randomized controlled trial assessed the efficacy of a clinic-based structured educational group incorporating psychological approaches to improve long-term glycemic control, quality of life and psychosocial functioning in children and adolescents with T1D. 28 pediatric diabetes services were randomized to deliver the intervention or standard care. 362 children (8–16 years) with HbA1c≥8.5% were recruited. Outcomes were HbA1c at 12 and 24 months, hypoglycemia, admissions, self-management skills, intervention compliance, emotional and behavioral adjustment, and quality of life. A process evaluation collected data from key stakeholder groups in order to evaluate the feasibility of delivering the intervention.

**Results:**

298/362 patients (82.3%) provided HbA1c at 12 months and 284/362 (78.5%) at 24 months. The intervention did not improve HbA1c at 12 months (intervention effect 0.11, 95% CI −0.28 to 0.50, p=0.584), or 24 months (intervention effect 0.03, 95% CI −0.36 to 0.41, p=0.891). There were no significant changes in remaining outcomes. 96/180 (53%) families in the intervention arm attended at least 1 module. The number of modules attended did not affect outcome. Reasons for low uptake included difficulties organizing groups and work and school commitments. Those with highest HbA1cs were less likely to attend. Mean cost of the intervention was £683 per child.

**Conclusions:**

Significant challenges in the delivery of a structured education intervention using psychological techniques to enhance engagement and behavior change delivered by diabetes nurses and dietitians in routine clinical practice were found. The intervention did not improve HbA1c in children and adolescents with poor control.

**Trial registration number:**

ISRCTN52537669, results.

**Key messages**

- The CASCADE intervention was not able to demonstrate improved diabetes control in children and adolescents with poor glycemic control when delivered in routine National Health Service (NHS) care by specialist clinic staff given 2 days standardized training.
- Despite evidence that the intervention was popular with staff and with families that attended, there were significant challenges in the organization and delivery of groups alongside standard practice that are often not acknowledged.
- Interventions need to be tailored to the specific needs of children and families and may need greater flexibility in delivery and more specialist staff. The challenges in the ability to deliver structured education and the failure to provide any measurable benefits to children and young people or their parents that received the intervention has important implications for clinical practice and national policy.

## Background

Type 1 diabetes (T1D) is a common chronic condition in which control of glycemia in childhood and adolescence is strongly predictive of later diabetic complications.^1^ Yet the majority of children and adolescents with T1D have poor glycemic control, even in high-income countries.^2^ In the UK <16% of children and adolescents have an glycated hemoglobin (HbA1c) below 7.5% (<58 mmol/mol), the recommended target for prevention of long-term complications,^3^ despite the widespread use of intensive insulin regimens and growing use of insulin pumps.

The ineffectiveness of intensive insulin regimens in improving population childhood diabetes control has focused attention on other factors including self-management skills, adherence behaviors and psychosocial adjustment.^4^ ^5^ A key health system factor identified as promoting good childhood diabetes control is the provision of diabetes education involving families.^6^ Structured standardized diabetes education programmes have been shown to improve glycemic control in adults with T1D.^7^ ^8^ Although delivery of a structured diabetes education programme is a requirement for specialist funding of pediatric diabetes services in England^9^ and in Germany, there is no evidence from randomized controlled trials to support the effectiveness of structured education for improving diabetes control in children and adolescents. Currently, standard care in UK clinics does not include any evidence-based structured education programmes.

In contrast, pilot studies suggest that motivational interviewing (MI) and solution-focused brief therapy (SF) show efficacy in reducing HbA1c, fear of hypoglycemia and improving quality of life (QOL) and positive well-being.^10–17^ We undertook a large cluster-randomized pragmatic trial of a structured education programme for child and adolescent diabetes that incorporated MI and SF components, funded by the National Institute of Health Research (NIHR) Health Technology Assessment Programme. The protocol has been previously reported.^18^

## Methods

### Intervention

The Child and Adolescent Structured Competencies Approach to Diabetes Education (CASCADE) intervention is a manual-based four-module structured education programme which uses solution focused and motivational approaches to increase engagement and enhance behaviour change in children, young people and families. The aim was for groups of three to four families to attend one module a month, over a 4-month period. The intervention was modified from a psychology-led clinical intervention which improved HbA1c by 1.5% (16 mmol/mol) in a non-randomized controlled pilot.^11^ Each module was designed to develop confidence in managing different aspects of diabetes, including how to adapt the amount of insulin taken, how to eat normally and how to manage daily challenges such as exercise and illness. The curriculum conformed to the agreed core content for education programmes set out by the T1D Education Network and Diabetes UK.

### Module 1: the relationship between food, insulin and blood glucose

Module 1 provides educators with notes about carbohydrate foods, blood glucose and a healthy diet, and international guidelines on nutritional management in childhood and adolescent diabetes.^19^ The introduction helps educators and families identify strengths, resources and abilities. Families identify how things will be in the future if they get what they want from CASCADE and scale how close they currently are to this. The session focuses on understanding different food groups, particularly carbohydrate. It talks about the role of insulin and different kinds of insulin regimens. Families and young people are encouraged to consider the pros and cons of matching insulin to food to attain better glycemic control.

### Module 2: blood glucose testing

Reading for module 2 are the implications of the Diabetes Care and Complications Trial (DCCT)^20^ and assessment and management of hypoglycemia in children and adolescents.^21^ After reviewing the previous module educators discuss the recommended target HbA1c. The group identifies factors which cause BG to rise and fall and explored how individuals define hypoglycemia, reviewing symptoms according to severity. Families discuss ways to treat hypoglycemia and assess the pros and cons of BG testing.

### Module 3: adjusting insulin—pros and cons

Reading for module 3 is on insulin analogs in diabetes care,^22^ using carbohydrate counting^23^ and guidelines on assessment and monitoring of glycemic control.^24^

Families discuss the symptoms of hyperglycemia and the significance of ketones. A brainstorming session considers when and how and who to contact for help managing hyperglycemia. The group focuses on managing high BG levels using temporary insulin changes and how to calculate correction doses. The group explores the advantages of identifying trends in relation to permanent insulin dose changes. The final exercise considers the advantages and disadvantages of carbohydrate counting as a way of improving glycemic control.

### Module 4: living with diabetes

Guidelines on exercise^25^ are provided with a set of training slides from the workshop to explain the principles of exercise and management of insulin and carbohydrates. Families identify the effect that low and high blood glucose levels have on performance and concentration and discuss strategies they already use to bring BG levels into target range before starting activity.

Family groups identify how different activities affect BG levels and discuss the timing of insulin injections in relation to activity. Young people discuss the advantages of using carbohydrate before, during and after exercise (to keep their BG stable during different kinds of activity).

At the end of module 4, young people and families complete a ‘blueprint for success’. This marks the end of the sessions and acknowledges the steps into the future the young person has already made. It creates an opportunity to review the programme and strengthens long-term motivation to change by reviewing previous successful goals.

The two main psychological approaches used in the delivery of CASCADE are MI and SF. Specific components of each approach were incorporated into each module to enhance engagement and develop confidence and motivation to change. The following techniques were integrated throughout each module. Communication skills: focusing on positive solutions: identifying skills abilities and strengths;^26^ encouraging families and children to identify previous successes; describing ongoing positive developments; focusing on the future; scaling; considering the pros and cons of behavior change; establishing the importance and confidence of change;^27^ using ‘scaffolding’ to help individuals discover information for themselves.^28^ Additional learning is a collaborative effort between individuals and trainers reducing the sense of an expert imposing knowledge and moving toward a shared venture. This active rather than passive approach has been shown to be effective in eliciting behavior change in other areas.^29^ The manual also included training on running groups that was presented in the workshops.

Two members (DC and RT) taught eight 2-day workshops that included MI and SF principles as well as the content and delivery of the four modules to a minimum of two educators per intervention site. Each site sent the required minimum of one pediatric clinical nurse specialist plus another member of the diabetes team with some sites sending more than two. A total of 43 staff attended over a number of weeks. Staff from a minimum of one clinic and a maximum of three were trained together in each workshop. A few sites found it challenging to ‘free up’ staff to attend. A detailed intervention manual and resources were provided.^30^ The training was designed to increase daily use of behavior change techniques and improve communication in healthcare encounters with patients as well as greater consideration of emotional as well as physical needs of young people and the social constraints of family life.

The intervention groups could be offered during standard clinic times or at different times of the day or week depending on what resources were available to the clinical teams. The educators were encouraged to be as flexible as possible within the remit of their job plans. Advice on organizing groups was also available from the research team. Most groups were offered in the clinic during normal clinic hours with a small number offered in a community space out of hours. Further details on the issues and challenges faced by clinic staff in intervention delivery are described in Christie *et al*.^30^

### Controls

Standard care was provided by the control sites. This involved regular clinic visits to normal clinics and appointments with nursing staff and other clinic staff as clinically indicated or requested by families. The specific number of appointments, who patients were seen by and how much contact was available varied significantly across sites.^30^

### Patients and trial design

We undertook a pragmatic cluster-randomized controlled trial with pediatric diabetes clinic as the unit of randomization with integral process and economic evaluations.^18^ Eligible clinics were National Health Service (NHS) pediatric diabetes services in London, the South East and Midlands of England, staffed by at least one pediatrician and pediatric nurse, which were not running group education at time of recruitment and had not taken part in a similar trial within the past 12 months.

Children and young people (8–16 years old) with T1D and a mean 12-month HbA1c of 8.5% (69 mmol/mol) or above were eligible to participate. Young people were excluded if they had (1) a significant learning disability or mental health problems unrelated to diabetes requiring mental health treatment, (2) significant other chronic illness in addition to diabetes and (3) insufficient command of English to enable full participation in the intervention.

Clinics wrote to the parent/guardian of all eligible patients before they were approached in clinic. Informed written consent or assent was obtained for participation from both the young person and parent/carer. Families were offered £10 at both the 12-month and 24-month data collection points as thanks for participation. The study was approved by the Research Ethics Committee of University College London Hospital.

### Outcomes collected

#### Primary outcome

The primary trial outcome was change in DCCT aligned HbA1c measured at baseline, 12 and 24 months after the baseline blood sample. Samples were sent to a single UK laboratory to ensure direct comparability of results from all clinics. Results were reported directly to the data manager following adjustment against the DCCT international standard.

#### Secondary outcomes

Specific versions of questionnaires were created for: 8–12, 13–16 years olds and carers with a modified version for those on pump therapy. Service user perspectives were sought on the structure and content of all the questionnaires. Demographic information and clinical data included years since diagnosis, insulin type, dose, number of injections and time at current clinic. Carer outcomes included demographic information (age, gender, ethnic origin, home ownership and deprivation score).

#### Psychosocial outcomes

Parental and self-report of health-related QOL was measured using the generic and diabetes module of the Pediatric Quality of Life Inventory (PedsQL).^31^ The generic module generates a psychosocial and physical health summary score. The psychosocial score is based on questions about emotional social and school functioning. The diabetes module generates five scores looking at problems with symptoms (diabetes) problems with injections, following the care plan or getting embarrassed (treatment I) carrying out diabetes-related tasks (treatment II) worrying about the effects of diabetes (worry) and communicating about diabetes. The five-item ‘Impact Supplement’ of the Strengths and Difficulties Questionnaire (SDQ; parent and child report versions) assessed the impact of identified emotional and behavioral difficulties on the young person's life.^32^ Responsibility for diabetes management was recorded using the Diabetes Family Responsibility Questionnaire (DFRQ).^33^ The number and severity of hypoglycemic episodes, hospital admissions, service utilization, clinic attendance, and contacts were collected through case note review after the 24-month data collection point.

#### Randomization of sites

Clinics were randomly allocated using a schedule drawn up by the Clinical Trial Unit at the London School of Hygiene and Tropical Medicine. Allocation was concealed until after clinics had consented and a first participant recruited to minimize selection biases at entry of clusters to the trial. Randomization was in a 1:1 ratio and minimized by factors likely to influence clinic mean HbA1c (ie, age of target population of the clinic (pediatric or adolescent), degree of clinic specialization (district general hospital clinic or teaching hospital/tertiary clinic) and size of clinic. Staff were requested not to inform families about randomization status until recruitment had finished. The patient's general practitioner was informed that their patient was taking part in a trial and which arm the hospital had been randomized to. The central laboratory assessing primary outcome (HbA1c, see below) remained blind to participant allocation.

#### Sample size

A sample size of 308 patients from 28 clinics would enable the trial to detect a difference between group HbA1c of 0.5 SDs, that is, 0.75% (8 mmol/mol) with 87% power at a significance level of 0.05 (two-tailed).

Our initial sample size calculation was based on a SD of Hb1Ac in the target population of ∼1.5%. (16 mmol/mol) We proposed to recruit sufficient young people to allow the trial to detect a difference between groups of 0.5 SDs, that is, 0.75% (8 mmol/mol) with 90% power at a significance level of 0.05 (two-tailed) and assumed an intracluster correlation coefficient (ICC) of 0.1. With these assumptions, 13 clinics in each arm with an average of 20 young people in each would be required. Given the possible loss to follow-up of ∼10%, the target recruitment was inflated to 22 young people from each clinic. However, based on initial within-clinic recruitment rates of 25% and lower than expected numbers of eligible patients per clinic, we revised our calculations based on recruiting 11 patients per clinic, and include the recruitment of two additional clinics. The revised calculations are based on an ICC (estimated from available baseline data (168 participants)) of 0.01.

#### Analyses

The primary analysis was an intention-to-treat comparison. All analyses of continuous outcomes used analysis of covariance to adjust for the baseline measure of the outcome in question and specified a random effect of clinic to account for clustering at that level. As most secondary outcomes were highly skewed, 95% CIs for these measures were estimated using 2000 bootstrap samples. For binary outcomes, mixed-effects logistic regression, adjusted for baseline measures where possible, was used to estimate the effect of the intervention. As there was no differential attrition by arm and no reason to believe the data were missing not at random, all analyses used complete case analysis.

Prespecified subgroup analyses included age (pediatric/adolescent), gender, high/low baseline levels of HbA1c (<10.4% (90 mmol/mol) vs ≥10.4% (90 mmol/mol)) and socioeconomic status (as measured by the multiple deprivation score) and effect sizes and 95% CIs estimated from models that included an interaction term.

A per protocol analysis of all primary and secondary outcomes was carried out with adherence to the protocol being defined as attending three or more of four CASCADE sessions. The same statistical analysis techniques were used as for the intent-to-treat analysis.

Serious adverse events (SAEs) were reported by participating sites; assessment of seriousness, causality and expectedness relating to the CASCADE intervention was carried out by a clinically trained member of the research team blind to allocation. SAEs were also recorded during the case note review.

The Data Monitoring and Ethics Committee confidentially reviewed unblinded interim analyses on three occasions and did not recommend stopping the trial early.

#### Process evaluation

A mixed-methods approach was used in the integral process evaluation. Questionnaires, semistructured interviews, non-participant informal discussion following observation sessions, fieldwork notes, and case note review were employed to collect qualitative and quantitative data from key stakeholder groups (University College London Hospital trainers, site educators, young people and parents) at specific time points in the trial.

The process evaluation was designed to assess the feasibility and acceptability and fidelity of delivering CASCADE groups as part of standard care within a NHS setting. Should the CASCADE intervention fail to be effective, the process evaluation was designed to assess the extent to which theory or implementation was responsible.

#### Economic analyses

The economic evaluation compared the CASCADE intervention to current NHS practice. Assessment of CASCADE cost-effectiveness with respect to current NHS practice focused on the cost of delivering the intervention and on the relative success controlling HbA1c and predicted impact on diabetic complications over time.^34^ The cost-effectiveness model considered long-term cost and benefit implications of delaying onset of microvascular and macrovascular diabetic complications by comparing HbA1c in the intervention and control groups.

## Results

**Figure 1:**
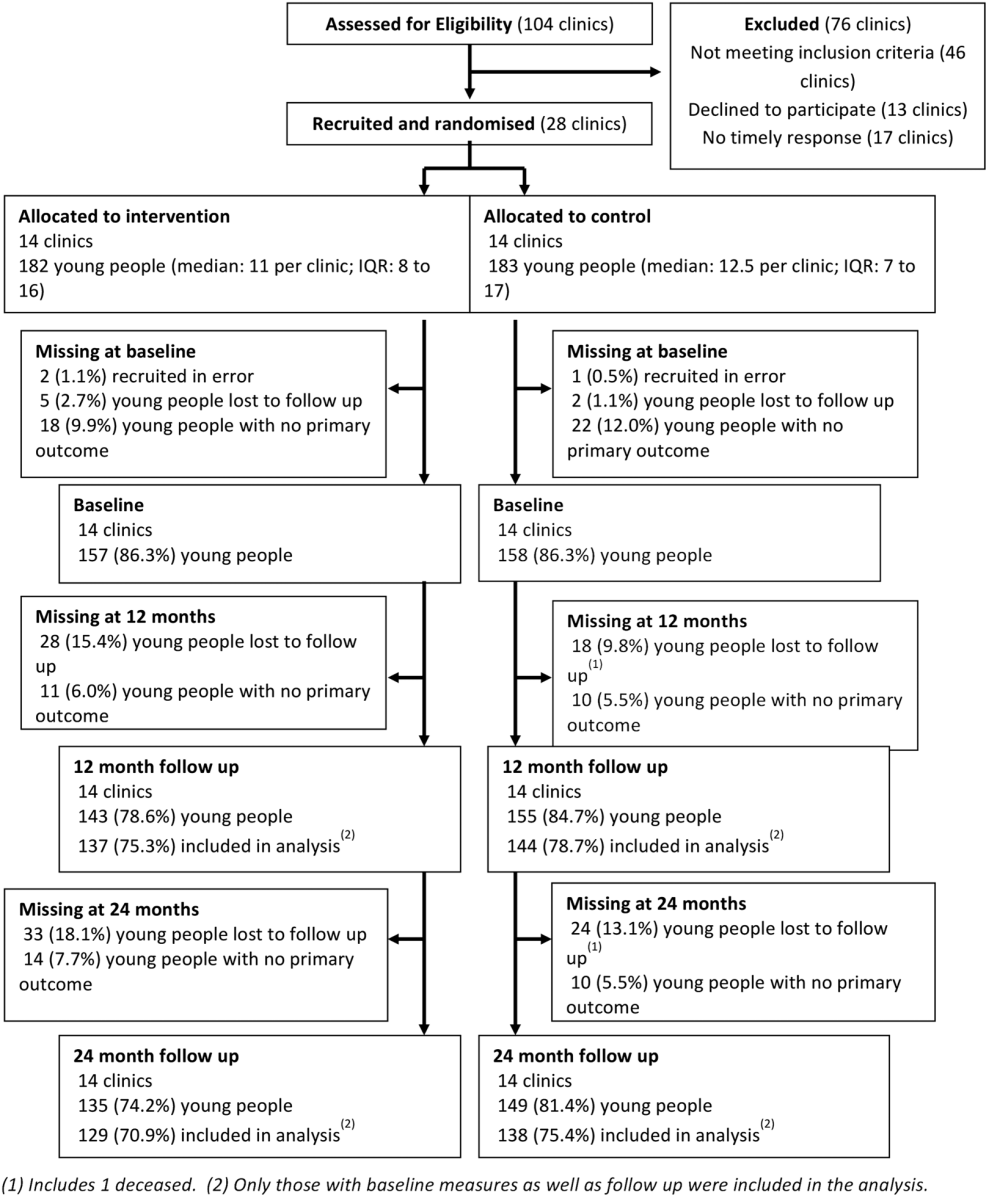
Consort diagram showing flow of clinics and young people through the trial.

In total, 1340 eligible patients were identified and sent information about the study; 1177 attended clinic appointments and were approached and invited to take part; 365 patients were recruited between February 2009 and September 2010, that is, 27% of those eligible. Reasons for declining to participate included lack of time and dislike of giving venous blood samples. Three patients were recruited with ineligible HbA1c levels and were excluded from further analysis, leaving 362 as the final sample. Figure 1 shows the flow of clinics and young people through the trial.

Demographic information on the 28 clinics and on the 327 children and young people and 324 parents who completed the baseline questionnaire is shown in table 1. The two groups were comparable at trial entry with all clinics mixed with regards to age; 86% in each arm were general hospital clinics with comparable numbers of eligible patients in each arm. Primary and secondary outcome measures outcomes by study arm at baseline are shown in table 2. The two arms appear well balanced at baseline for all outcomes. We did not test for baseline differences as we comply with CONSORT guidelines^35^ that advise against statistical testing of differences between randomized groups that are most likely caused by chance.

**Table 1:** Baseline characteristics of clinics, young people and parents/carers

	Intervention	Control
Clinics	N=14	N=14
Age profile of patients*		
Pediatric (0–12); n (%)	0	0
Adolescent (13–18); n (%)	0	0
Mixed; n (%)	14 (100)	14 (100)
Type of clinic*		
General hospital; n (%)	12 (86)	12 (86)
Specialist/tertiary; n (%)	2 (14)	2 (14)
Number of eligible patients**		
≤32	6 (43)	4 (28)
33 to 50	5 (36)	5 (36)
>50	3 (21)	5 (36)
Size of clinic		
Number attending clinic; mean (SD)	122 (45.8)	140 (51.3)
More than 50 young people registered; n (%)	14 (100)	14 (100)
More than 100 young people registered; n (%)	11 (79)	9 (64)
Staffing		
f/t DSN or equiv; n (%)	14 (100)	14 (100)
Dietician support; n (%)	13 (93)	13 (93)
Young people	N=159	N=168
Gender		
Female; n (%)	91 (57.2)	90 (53.6)
Male; n (%)	68 (42.8)	78 (46.4)
Age (years); mean (SD)	13.1 (2.1)	13.2 (2.1)
Ethnicity		
White British; n (%)	133 (83.7)	129 (76.8)
White other; n (%)	5 (3.1)	5 (3.0)
Mixed; n (%)	7 (4.4)	4 (2.4)
Asian/Asian British; n (%)	5 (3.1)	14 (8.3)
Black/black British; n (%)	5 (3.1)	6 (3.6)
Chinese; n (%)	0	0
Other; n (%)	4 (2.5)	9 (5.4)
Time since diagnosis (years); mean (SD)	5.7 (3.2)	6.1 (3.3)
Time since enrolled at participating clinic (years); mean (SD)	5.1 (2.9)	5.6 (3.2)
Parents/carers	N=156	N=169
Gender		
Female; n (%)	137 (87.8)	151 (89.4)
Male; n (%)	19 (12.2)	17 (10.1)
Relationship to young person with diabetes		
Mother; n (%)	136 (87.2)	153 (90.5)
Father; n (%)	17 (10.9)	13 (7.7)
Female guardian; n (%)	0	1 (0.6)
Male guardian; n (%)	0	0
Other; n (%)	2 (1.3)	0
Partnership status		
Married; n (%)	89 (57.1)	113 (66.9)
Cohabiting: n (%)	18 (11.5)	18 (10.7)
Single parent; n (%)	45 (28.9)	36 (21.3)
Ethnicity		
White British; n (%)	135 (86.5)	132 (78.1)
White other; n (%)	7 (4.5)	5 (3.0)
Mixed; n (%)	0	2 (1.2)
Asian/Asian British; n (%)	6 (3.9)	15 (8.9)
Black/black British; n (%)	4 (2.6)	5 (3.0)
Chinese; n (%)	1 (0.6)	1 (0.6)
Other; n (%)	3 (1.9)	8 (4.7)
Tenure		
Privately owned; n (%)	106 (68.0)	98 (58.0)
Council/rented; n (%)	48 (30.8)	68 (40.2)
Deprivation score mean (SD)	21.01 (13.1)	21.98 (15.58)

**Table 2:** Diabetes knowledge, confidence and management at baseline

	Young person		Parent/carer	
	Intervention N=159	Control N=168	Intervention N=156	Control N=169
*Management*				
HbA1c (venepuncture)				
Mean (%) (SD)	9.9% (1.5)	10.0% (1.5)	–	–
Mmol/mol mean (SD)	85 mmol/mol (16)	86 mmol/mol (16)		
Number of severe hypoglycemic episodes in last month; n (%)*				
1	12 (7.6)	13 (7.8)		
2	2 (1.3)	2 (1.2)		
3	1 (0.6)	1 (0.6)		
12	1 (0.6)	0		
Number of times admitted in past 6 months; n (%)*				
1	23 (14.7)	16 (9.5)		
2	0	2 (1.2)		
3	1 (0.6)	1 (0.6)		
10	1 (0.6)	0		
Number of times attended diabetes clinic in last year; median (IQR)	4.0 (3.5, 4.0)	4.0 (3.5, 4.0)		
Visit hospital doctor most/every visit; n (%)	130 (89.0)	142 (88.8)		
Visit diabetes nurse most/every visit; n (%)	139 (92.1)	158 (95.8)		
Visit dietician occasionally or more often; n (%)	129 (92.1)	129 (84.9)		
Visit psychologist occasionally or more often; n (%)	16 (14.7)	19 (15.5)		
*PedsQL—general; median (IQR)*				
Physical health summary score	90.6 (84.4, 96.9)	90.6 (81.3, 96.9)	89.1 (78.6, 93.8)	87.5 (78.1, 96.9)
Psychosocial health summary score	83.9 (75.0, 91.7)	81.7 (70.0, 90.0)	76.7 (63.8, 86.7)	75.0 (61.7, 85.0)
Total score	87.0 (76.1, 92.4)	84.8 (75.0, 91.3)	79.3 (70.1, 89.1)	80.4 (68.5, 88.0)
*PedsQL—diabetes module; median (IQR)*				
Diabetes score	62.5 (50.0, 77.3)	61.4 (50.0, 72.7)	56.8 (45.5, 68.2)	54.5 (43.2, 65.9)
Treatment 1 score	75.0 (62.5, 87.5)	81.3 (62.5, 93.8)	62.5 (43.8, 75.0)	62.5 (50.0, 75.0)
Treatment 2 score	85.7 (75.0, 92.9)	87.5 (71.4, 96.4)	78.6 (65.0, 89.3)	75.0 (64.3, 91.7)
Worry score	75.0 (50.0, 91.7)	75.0 (58.3, 91.7)	66.7 (50.0, 83.3)	66.7 (50.0, 83.3)
Communication score	75.0 (50.0, 91.7)	83.3 (66.7, 100.0)	66.7 (50.0, 100.0)	75.0 (50.0, 100.0)
*Diabetes family responsibility questionnaire; median (IQR)*				
Family responsibility total score	35.0 (31.0, 38.3)	36.0 (32.0, 39.0)	31.0 (27.5, 35.0)	31.8 (28.0, 35.0)
*Strengths and difficulties questionnaire; median (IQR)*				
Strengths and difficulties total average impact score	0.0 (0.0, 0.0)	0.0 (0.0, 0.0)	0.0 (0.0, 0.0)	0.0 (0.0, 0.0)
Normal; n (%)	139 (89.1)	145 (89.0)	126 (81.8)	138 (82.1)
Borderline; n (%)	2 (1.3)	3 (1.8)	4 (2.6)	5 (3.0)
Abnormal; n (%)	15 (9.6)	15 (9.2)	24 (15.6)	25 (14.9)
*Body weight and insulin; median (IQR)*				
Happiness with body weight	8.0 (5.0, 9.0)	7.0 (4.0, 9.0)	–	–
Number of times skipped insulin in last month; median (IQR)	0 (0, 2)	0 (0, 2)	–	–
Ever skipped insulin to lose weight; n (%)	8 (5.0)	9 (5.4)	–	–

*Data provided by case note review.

PedsQL, Pediatric Quality of Life Inventory.

Ninety-six of the 180 young people recruited to the intervention arm (53%) attended at least one CASCADE module. There were no significant differences in attendance by gender, duration of diabetes, ethnicity or level of deprivation in the area in which they lived. Eighty-four young people (47%) failed to attend any modules. Seven (4%) dropped out of the study before the CASCADE modules were offered in their clinic, 11 (6%) were never offered modules to attend, and 66 (37%) were offered the opportunity to attend the modules, but opted not to do so. The main reason for opting out was desire not to miss school or other activities.

The primary outcomes at 12 and 24 months are shown in table 3. The mean HbA1c at 12 months was 10.2% (88 mmol/mol) in the intervention group and 10.1% (87 mmol/mol) in the control group with an estimated adjusted mean difference of 0.11 (95% CI −0.28 to 0.50). The ICC for the primary outcome at 12 months was 0.134 and 0.060 at 24 months.

**Table 3:** HbA1c from venepuncture at 12 and 24 months follow-up

	Intervention		Control		Intervention effect
	N	Mean (SD)	N	Mean (SD)	Estimate (95% CI) p Value
12-month follow-up					
HbA1c (%) at 12 months Mmol/mol (SD)	143	10.2% (2.0) 88 mmol/mol (21)	155	10.1% (1.6) 87 mmol/mol (17)	0.11 (−0.28 to 0.50) p=0.584
Change in HbA1c at 12 months from baseline	137	0.38 (1.34)	144	0.28 (1.27)	
24-month follow-up					
HbA1c (%) at 24 months	135	10.1% (1.9) 87 mmol/mol (20)	149	10.0% (1.7) 86 mmol/mol (18)	0.03 (−0.36 to 0.41) p=0.891
Change in HbA1c at 24 months from baseline	129	0.10 (1.52)	138	0.07 (1.53)	

HbA1c, glycated hemoglobin.

The secondary outcomes as reported by the participants and their carers at 12 and 24 months are shown in tables 4 and 5. Outcomes were similar between the two arms of the trial.

**Table 4:** Diabetes knowledge, confidence and management at 12 months follow-up

	Young person			Parent/carer		
	Intervention	Control	Intervention	Intervention	Control	Intervention
	Median (IQR)	Median (IQR)	Effect (95% CI)	Median (IQR)	Median (IQR)	Effect (95% CI)
Regimen						
Number of injections per day	4.0 (4.0, 4.0)	4.0 (4.0, 4.0)	0.08 (−0.22 to 0.34)			
Average total dose of quick acting insulin per day	30.0 (22.0, 42.0)	25.5 (16.0, 37.0)	5.06 (1.04 to 9.93)			
Average total dose of slow acting insulin per day	30.0 (22.0, 42.0)	26.5 (15.5, 36.0)	4.94 (0.61 to 9.16)			
*Management*						
Number of severe hypoglycemic episodes in last month; n (%)						
1	9 (6.3)	11 (7.1)	0.76* (0.35 to 1.67)			
2	2 (1.4)	2 (1.3)				
3	1 (0.7)	1 (0.7)				
4	0	1 (0.7)				
5	0	1 (0.7)				
Number of times admitted in past 6 months; n (%)						
1	17 (11.9)	14 (9.0)	1.08* (0.54 to 2.14)			
2	2 (1.4)	2 (1.3)				
6	0	1 (0.7)				
8	0	1 (0.7)				
Number of times attended diabetes clinic in last year	4.0 (3.5, 4.0)	4.0 (3.5, 4.0)	0.01 (−0.22 to 0.24)			
Visit hospital doctor most/every visit; n (%)	127 (93.4)	127 (90.1)	2.71 (0.81 to 9.05)			
Visit diabetes nurse most/every visit; n (%)	128 (91.4)	143 (95.3)	0.68 (0.22 to 2.05)			
Visit dietician occasionally or more often; n (%)	121 (91.7)	122 (90.37)	1.64 (0.53 to 5.06)			
Visit psychologist occasionally or more often; n (%)	19 (20.2)	12 (11.0)	3.64 (0.56 to 23.63)			
*Pedsq1—general; median (IQR)*						
Physical health summary score	90.6 (84.4, 96.9)	88.4 (78.1, 96.9)	0.34 (−2.51 to 2.62)	90.6 (81.3, 96.9)	87.5 (71.9, 93.8)	2.24 (−1.34 to 5.10)
Psychosocial health summary score	81.7 (70.0, 88.3)	81.7 (71.4, 90.0)	−1.85 (−4.29 to 0.24)	78.3 (63.3, 88.3)	71.7 (58.3, 83.3)	1.76 (−1.50 to 5.79)
Total score	83.7 (76.1, 90.2)	82.6 (75.0, 89.1)	−1.09 (−3.15 to 0.63)	82.6 (70.7, 89.1)	77.2 (63.0, 85.9)	1.74 (−1.14 to 5.00)
*PedsQL—diabetes module; median (IQR)*						
Diabetes score	61.4 (51.1, 72.7)	61.4 (50.0, 70.5)	0.62 (−2.35 to 3.04)	56.8 (50.0, 70.5)	54.5 (40.9, 65.9)	1.13 (−1.72 to 4.02)
Treatment 1 score	75.0 (56.3, 87.5)	81.3 (62.5, 93.8)	−0.80 (−5.14 to 3.08)	62.5 (50.0, 81.3)	62.5 (50.0, 81.3)	0.79 (−2.58 to 4.10)
Treatment 2 score	85.7 (75.0, 92.9)	87.5 (75.0, 96.4)	−1.90 (−4.99 to 1.97)	82.1 (64.3, 92.9)	75.0 (62.5, 89.3)	2.37 (−0.40 to 5.37)
Worry score	75.0 (58.3, 91.7)	75.0 (58.3, 91.7)	−0.77 (−5.43 to 3.94)	75.0 (50.0, 91.7)	66.7 (50.0, 83.3)	3.59 (−1.50 to 7.57)
Communication score	75.0 (58.3, 91.7)	79.2 (58.3, 91.7)	−1.34 (−6.31 to 4.01)	75.0 (50.0, 100.0)	75.0 (50.0, 100.0)	2.30 (−2.44 to 8.64)
*Diabetes family responsibility questionnaire; median (IQR)*						
Family responsibility total score—young person (weighted*†)	36.0 (32.9, 39.0)	36.0 (32.6, 40.0)	0.01 (−0.85 to 0.81)	33.0 (29.0, 36.0)	32.5 (29.0, 36.0)	0.62 (−0.17 to 1.47)
Dyadic parent–child responsibility pattern	–	–	–	0 (0, 1)	0 (0, 1)	0.00 (−0.22 to 0.20)
*Strengths and difficulties questionnaire; median (IQR)*						
Strengths and difficulties total average impact score	0.0 (0.0, 0.0)	0.0 (0.0, 0.0)	0.09 (−0.21 to 0.43)	0.0 (0.0, 0.0)	0.0 (0.0, 0.0)	−0.03 (−0.31 to 0.27)
Normal; n (%)	130 (87.8)	148 (93.7)	–	118 (83.7)	127 (82.5)	–
Borderline; n (%)	1 (0.7)	3 (1.9)		5 (3.6)	5 (3.3)	
Abnormal; n (%)	17 (11.5)	7 (4.4)		18 (12.8)	22 (14.3)	
*Body weight and insulin; median (IQR)*						
Happiness with body weight	7.0 (4.0, 9.0)	7.0 (4.0, 9.0)	−0.24 (−0.65 to 0.29)	–	–	–
Number of times skipped insulin in last month	0 (0, 2)	0 (0.3)	0.82* (0.48 to 1.38)	–	–	–
Ever skipped insulin to lose weight; n (%)	4 (2.7)	11 (6.9)	0.28 (0.07 to 1.13)	–	–	–

*OR for once or more versus none.

†Weighted to allow for non-response.

PedsQL, Pediatric Quality of Life Inventory.

**Table 5:** Diabetes knowledge, confidence and management at 24 months follow-up

	Young person			Parent/carer		
	Intervention N=144	Control N=151	Intervention effect	Intervention N=137	Control N=140	Intervention effect
	Median (IQR)	Median (IQR)	Estimate effect (95% CI)	Median (IQR)	Median (IQR)	Estimate effect (95% CI)
Regimen						
Number of injections per day	4.0 (4.0, 4.0)	4.0 (4.0, 4.0)	−0.14 (−0.43 to 0.15)			
Average total dose of quick acting insulin per day	34.0 (25.0, 50.0)	30.0 (18.0, 40.0)	6.87 (1.26 to 12.57)			
Average total dose of slow acting insulin per day	29.0 (22.0, 42.0)	27.5 (19.0, 34.5)	5.85 (1.58 to 9.73)			
*Management*						
Number of severe hypoglycemic episodes in last month; n (%)						
1	6 (4.4)	7 (5.0)	0.92* (0.32 to 2.59			
2	1 (0.7)	3 (2.1)				
3	3 (2.2)	1 (0.7)				
Number of times admitted in past 6 months; n (%)						
1	11 (8.0)	12 (8.6)	0.95* (0.46 to 1.96)			
2	2 (1.5)	3 (2.1)				
3	2 (1.5)	1 (0.7)				
4	1 (0.7)	0 (0.0)				
6	0 (0.0)	1 (0.7)				
Number of times attended diabetes clinic in last year	4.0 (3.0, 4.0)	4.0 (3.5, 4.0)	−0.18 (−0.41 to 0.06)			
Visit hospital doctor most/every visit; n (%)	121 (93.8)	115 (85.8)	3.09 (1.07 to 8.91)			
Visit diabetes nurse most/every visit; n (%)	125 (94.7)	122 (90.4)	2.70 (0.92 to 7.92)			
Visit dietician occasionally or more often; n (%)	118 (92.2)	106 (85.5)	2.26 (0.86 to 5.93)			
Visit psychologist occasionally or more often; n (%)	27 (25.5)	14 (12.8)	3.85 (0.77 to 19.31)			
*PedsQL—general*						
Physical health summary score	90.6 (81.3, 96.9)	87.5 (81.3, 96.9)	1.14 (−1.28 to 3.32)	87.5 (81.3, 93.8)	87.5 (71.9, 93.8)	2.00 (−2.04 to 5.29)
Psychosocial health summary score	80.0 (68.3, 88.3)	81.7 (71.7, 88.3)	−1.17 (−3.69 to 1.45)	75.0 (63.3, 88.3)	73.3 (61.7, 84.2)	0.03 (−3.08 to 3.66)
Total score	83.7 (75.0, 90.2)	84.2 (75.0, 90.2)	−0.33 (−2.53 to 1.97)	77.2 (69.6, 90.2)	77.2 (66.3, 87.0)	0.58 (−2.70 to 3.46)
*PedsQL—diabetes module*						
Diabetes score	63.6 (50.0, 75.0)	63.6 (54.5, 75.0)	−0.02 (−3.19 to 2.72)	56.8 (47.7, 68.2)	56.8 (45.5, 71.6)	−0.08 (−2.83 to 3.12)
Treatment 1 score	75.0 (62.5, 87.5)	81.3 (62.5, 93.8)	−1.05 (−4.52 to 2.32)	68.8 (50.0, 81.3)	62.5 (50.0, 81.3)	2.20 (−2.31 to 6.88)
Treatment 2 score	85.7 (71.4, 95.8)	85.7 (75.0, 95.8)	−1.49 (−4.53 to 1.42)	78.6 (64.3, 89.3)	78.6 (64.3, 92.9)	−1.10 (−4.49 to 2.50)
Worry score	75.0 (58.3, 87.5)	75.0 (58.3, 91.7)	−0.32 (−4.64 to 4.52)	66.7 (50.0, 83.3)	66.7 (50.0, 89.6)	0.66 (−3.75 to 5.14)
Communication score	83.3 (58.3, 91.7)	83.3 (66.7, 100.0)	−1.06 (−5.34 to 3.59)	75.0 (50.0, 100.0)	83.3 (50.0, 100.0)	−2.54 (−9.06 to 4.16)
*Diabetes family responsibility questionnaire*						
Family responsibility total score—young person (weighted *†)	38.0 (34.0, 41.0)	37.0 (34.0, 42.0)	0.71 (−0.07 to 1.44)	35.0 (31.9, 38.0)	34.0 (30.0, 38.5)	0.60 (−0.43 to 1.47)
Dyadic parent–child responsibility pattern	–	–	–	0 (0, 1)	0 (0, 1)	−0.03 (−0.26 to 0.23)
*Strengths and difficulties questionnaire*						
Strengths and difficulties total average impact score	0.0 (0.0, 0.0)	0.0 (0.0, 0.0)	0.04 (−0.14 to 0.21)	0.0 (0.0, 0.0)	0.0 (0.0, 0.0)	−0.09 (−0.41 to 0.26)
Normal; n (%)	130 (91.6)	135 (89.4)	–	110 (82.7)	107 (77.0)	–
Borderline; n (%)	2 (1.4)	4 (2.7)		2 (1.5)	6 (4.3)	
Abnormal; n (%)	10 (7.0)	12 (8.0)		21 (15.8)	26 (18.7)	
*Body weight and insulin*						
Happiness with body weight	7.0 (4.0, 9.0)	7.0 (5.0, 9.0)	−0.56 (−1.03 to −0.06)	–	–	–
Number of times skipped insulin in last month	0 (0, 2)	0 (0, 2)	1.30* (0.78 to 2.17)	–	–	–
Ever skipped insulin to lose weight; n (%)	5 (3.5)	7 (4.6)	0.72 (0.22 to 2.33)	–	–	–

*OR for once or more versus none.

†Weighted to allow for non-response.

PedsQL, Pediatric Quality of Life Inventory.

A per protocol analysis was performed including only intervention participants who had attended 3–4 of the 4 modules (n=55). This also found no difference in HbA1c between arms.

A number of prespecified subgroup analyses were carried out. The analysis of baseline HbA1c suggested that among those with HbA1c levels ≥10.4% (90 mmol/mol) at baseline, HbA1c levels at follow-up were greater in the intervention than the control arm, suggesting that those with higher HbA1c responded more poorly to the CASCADE intervention. This difference was statistically significant at 12 months but not at 24 months. There was no evidence that results differed according to age, gender or deprivation score

### Serious adverse events

Seven SAEs were reported in the intervention arm and 13 in the control arm during the study period. Despite reporting procedures being reviewed during the trial, it was recognized that fewer than expected SAEs were being reported. The case note re view data reported a total of 145 SAEs in the intervention arm and 187 in the control arm.

### Process evaluation

In every site, it proved feasible to train nurses and dieticians to be site educators; however, staff found organizing the groups burdensome in terms of arranging suitable dates/times and creating satisfactory group composition in the absence of dedicated administration time and skills. As a result, the intervention was not delivered to a large proportion of participant patients. Only 12 of the 14 sites delivered at least one complete group of four modules. Only 68% of possible groups were run. This was due to poor uptake from families after recruitment and baseline data collection and those groups that did run were smaller in size and more mixed in terms of ages of participants than intended. Delivering the intervention to these non-ideal groups was challenging. Take up was particularly low for those young people with the highest HbA1c. Those who attended had significantly lower mean baseline HbA1c scores than those who did not attend (9.52% (81 mol/mol) vs 10.33% (89 mmol/mol), p<0.01).

Of the 180 families in the intervention group, only 55 (30%) received the full programme with 53% attending at least one module. Significantly more children (8–12 years) attended at least one module compared with teenagers (13–16 years; 64% vs 44%, p<0.01).

Young people and parents who attended and staff who delivered CASCADE were enthusiastic about its relevance and members of all stakeholder groups reported some perceived impact. Parents and young people who attended described improvements in family relationships, knowledge and understanding, greater confidence and increased motivation to manage diabetes. At 24 months nearly half of the young people reported the groups had made them want to try harder with their diabetes and that they had carried on trying.

Fidelity of content delivery was moderately good, although consistent use of motivational techniques was challenging for some site educators. In two sites, staff that had undergone training left and were replaced by staff who had not attended the workshops.

Implementation and model factors were further compounded by trial factors—including a smaller than expected numbers of recruits in some sites and a longer than planned for time lapse between training and delivery caused by delays in patient recruitment. For a longer discussion of these issues see Sawtell *et al*.^36^

### Economic analysis

The mean cost of providing the intervention was £5098 per site or £683 per child. The lifetime costs of the CASCADE intervention were estimated to be £216 greater than those of current practice.^30^ Since there was no difference in health outcome between the CASCADE intervention and current practice, the option with the lowest cost is the most cost-effective.

## Discussion

We evaluated current best practice in pediatric diabetes structured education with an additional psychological component designed to increase engagement and behavior change in routine care, using robust methodology and a diverse population. Despite evidence that the intervention was popular with staff and with families that attended, there were challenges in the organization and delivery of groups alongside standard practice that are often not acknowledged and as a consequence the intervention was not delivered to a large proportion of participant patients. The provision of structured education through the CASCADE intervention delivered in routine NHS care by specialist clinic staff given 2 days standardized training failed to demonstrate improved diabetes control in children and adolescents with poor glycemic control.

Our findings may reflect a lack of effectiveness of the CASCADE intervention or a lack of intervention ‘reach’ in this pragmatic trial, possibly due to multiple factors. Despite extensive efforts by the intervention clinics to offer groups at times that suited young people and families non-response and failure to attend rates were high. Only 53% of participants in the intervention arm received any intervention sessions. In the main, delivery issues were pragmatic issues related to lack of administrative support for intervention delivery within routine clinical services. One clinic was only able to deliver one out of the four group sessions and one clinic failed to deliver any groups. Second, the intervention was delivered by clinic nurses and dietitians after a relatively brief training, whereas in the pilot delivery was by qualified psychologists working alongside senior nursing staff. The varied fidelity in delivery of content and in particular specific motivational techniques suggests the training was insufficient for the skill level of the staff involved. Other studies have shown that nurses and dietitians can be successfully trained to use MI with nurses seeing this approach as consistent with their values and better than traditional advice-giving approaches.^37^

MI can appear to be a deceptively simple approach; however, while some of the concepts are straightforward to grasp intellectually, the skilful practice of MI takes time, effort and responsiveness to regular feedback as well as requiring appropriate training. Difficulty in delivery of key motivational components also suggests there was a need for ongoing supervision to ensure quality of delivery to families. In some cases there was also a failure to deliver the interventions as recommended. One site used an untrained member of staff as the second trainer in one module (and no second trainer in the other modules)—another site had partially trained staff ‘supporting’ sessions despite clear recommendation that training in delivery was necessary. Finally, the process evaluation raised issues about questions about timing (the intervention may have been more effective closer to diagnosis) and targeting. It might be argued that the intervention may have been more effective if aimed at with children with lower HbA1c and earlier in their diabetes history.^36^

The negative outcome of this trial must be examined in the context of other recent negative trials of psychosocial interventions in pediatric diabetes in the UK. Common characteristics of these trials (including CASCADE) were that (1) each offered a single intervention to improve glycemic control, and (2) each attempted to extend the role of existing diabetes nursing and medical staff to either deliver psychologically based interventions or deliver elements of routine care in ways informed by psychological theories such as MI. Together these trials suggest strongly that such approaches may be ineffective in improving diabetes control, particularly where control is already poor.^38–41^ Our findings add to this by suggesting that attempting to deliver psychologically informed structured education is not a useful remedy for poor diabetes control in children and adolescents if offered in routine clinical practice by diabetes specialist nurses or dietitians. It is important to note that our trial did not assess the value of routine structured education in newly diagnosed diabetes, and it is imperative that our findings are not generalized to those newly diagnosed.

## Conclusions

Significant challenges were encountered in the delivery of a structured education intervention using psychological techniques by diabetes nurses and dietitians in routine clinical practice. The intervention did not improve HbA1c in children and adolescents with poor control. Our findings suggest that the provision of structured education in pediatric diabetes care, now mandated in the UK and a requisite for insurance payment in Germany will not improve diabetic control in those with poor control. However, structured education should remain as a basic pre-requisite for care of all children and young people newly diagnosed with diabetes, with further attention given to stepped-care models, in which interventions tailored to the specific needs of the child and family are delivered by more specialist staff.
